# Spatial analysis of the death associated factors due oral cancer in Brazil: an ecological study

**DOI:** 10.1186/s12903-018-0473-y

**Published:** 2018-01-23

**Authors:** Gisele Pedroso Moi, Ageo Mário Cândido Silva, Noemi Dreyer Galvão, Marcelo de Castro Meneghim, Antonio Carlos Pereira

**Affiliations:** 1 Piracicaba Dental School, Campinas State University - FOP.UNICAMP, Piracicaba, Brazil; 2 Institute of Public Health, Federal University of Mato Grosso - ISC.UFMT, Cuiabá, Brazil; 3 University Center of Várzea Grande - UNIVAG, Várzea Grande, Brazil; 4 Rua Nossa Senhora da Guia, 504. Apto. 901.2, Jardim Santa Martha, Cuiabá, MT 78043-605 Brazil

**Keywords:** Mouth Neoplasms, Spatial analysis, Mortality, Epidemiology, Ecological studies, Risk factors

## Abstract

**Background:**

Oral cancer (OC) is among the ten most common cancers and the seventh most frequent cause of death worldwide. It has been reported that these incidence rates are higher in developed country and these mortality rates are higher in less developed areas. So, the objective of the present study was to analyze the spatial joint distribution and to explore possible associations of the epidemiological aspects with mortality rates due to OC in the Brazil.

**Methods:**

An exploratory ecological study investigated the global spatial autocorrelation of epidemiological aspects with mortality rates due to OC from the Brazilian Federative Units (FUs) (*n* = 27) in the period 2005–2014, using the “global” and “local” Moran statistic method and a multiple spatial regression, having as variables of exposure the habits and lifestyle, sociodemographic indicators, the consumption of pesticides, the presence of comorbidities, the use of health services and food consumption; and, as a variable response, mortality rates due to OC. The software used was Stata 11.0, SPSS 18.0 and GeoDa 0.95-i.

**Results:**

The spatial distribution of OC mortality rates to age-standard was not random and showed high spatial autocorrelation and predominance of significant spatial groupings in the Central-South region of Brazil. In the multiple regression, statistically negative associations were observed between the Human Development Index (HDI) and OC age-standardized in the studied period (*p* < 0.05) and positive associations among the proportion of the population with dental appointment within last year, percentage of consumption of oils and fats, percentage of consumption of ready-to-eat foods and industrial mixtures and percentage of overweight adults with this type of cancer (*p* < 0.05).

**Conclusion:**

This is the first study that analyzed the factors associated to the spatial clusters of mortality due to oral cancer in the Brazilian FUs. A fairly unequal distribution of OC mortality rates was found, being that these rates presented inverse association with HDI and direct association with dental appointment, consumption of oils and fats, ready-to-eat foods and industrial mixtures consumption and overweight these rates. It suggests the need to redirect Brazilian public policies aimed at combating them so that they cease to be temporary and become permanent.

## Background

Oral cancer (OC) is among the ten most common cancers and the seventh most frequent cause of death worldwide [1]. Its incidence increases with age, in countries with a high incidence of this malignant neoplasm in the worldwide, many cases are reported in young adults < 45 years [2].

The International Classification of Diseases for Oncology of possible sites for malignant neoplasms is accepted by World Health Organization and defines the sites belonging to OC with those involving the topographic regions of the lip, oral cavity and pharynx [3]. This pathology has its development stimulated by the interaction of environmental, behavioral and occupational factors (carcinogens or carcinogens) and factors related to the host itself (age, race, sex, spontaneous mutations and inherited mutations) [4]. Exposures to carcinogens are in some way related to socioeconomic inequalities and may contribute significantly to the incidence and mortality of cancer [5]. More than 80% of OC cases occurring in Western countries are associated with tobacco use and alcohol consumption [6]. Additionally, studies have also demonstrated that a poor diet plays a relevant role in the etiology of this pathology [6]. Besides, protective effects of a healthy diet have been observed and they are associated with a diet rich in fruits, vegetables, regular coffee consumption and regular intake of folic acid in the diet [7]. There are also other factors that may increase the risk of this malignant neoplasm, as well as exposure to pesticides, combustion gases, biomass burning, human papillomavirus (HPV) exposure and sun exposure [7, 8].

Estimates and predictions of cancer incidence and mortality are extremely relevant for the public health strategy planning process [9]. These provide references to exposure to risk factors and allow assessment of the effectiveness of existing health interventions (prevention campaigns, screening and treatment programs), making them valuable tools to redirect control programs and better distribute financial resources and humans [10]. However, these estimates alone are not sufficient to understand the true magnitude, trends of malignant neoplasms, and to evaluate interventions against cancer [11]. Thus, the objective of this study was to analyze the spatial joint distribution (see methodology) and to explore possible associations of the epidemiological aspects with mortality rates due to OC in the Brazil.

## Methods

This exploratory ecological study, approved by Research Ethics Committee of the University Center of Várzea Grande (CAAE: 62,746,616.1.0000.5692), included secondary data openly available from Brazilian population.

Brazil is a continental country and the most populous country in Latin America as well as one of the most populous in the world, has a surface area of 8,511,960 km^2^ and with a population of 190,755,799, being divided into five geographic regions (South, Southeast, Central-West, North and Northeast) and 27 Federative Units (26 states and one Federal District) [12].

The geographic and spatial data of each of Brazilian Federative Units (FUs), considering the latitude, longitude, perimeter, area and location of their capitals, were obtained from Brazilian Institute of Geography and Statistics (IBGE) website [13].

The dependent variable of this study was determined from the mortality rates for oral cancer (OC) from individuals aged ≥40 years at a Brazilian FUs in the period 2005–2014. It’s important to highlight that in this study the malignant neoplasm that affected the topographic regions of the lip, oral cavity and pharynx defined the term “oral cancer (OC)”, according to the International Classification of Diseases for Oncology and accepted World Health Organization [3]. The crude data of mortality from OC in individuals aged ≥40 years were selected in the Mortality Information System of the Ministry of Health from the deaths with a basic cause coded as C00-C14, in Chapter II of the 10th Revision of the ICD, according to distribution by Brazilian FUs [14]. The data of the total population resident in the Brazilian FUs during the study period were based on the 2010 population census and estimates for the years among census that were obtained from IBGE website [13]. The mortality rates from OC were calculated from the division of mortality data from OC in individuals aged ≥40 years in each Brazilian FUs by the data of the total population resident in the same place. The independent variables selected for this study was obtained from several databases of national scope in the period studied and are described in detail below:

- The *percentage of current tobacco consumption*, the *percentage overweight* (BMI ≥25 kg / m^2^) and *obesity* (BMI ≥30 kg / m^2^) was recovered from a national survey of risk factors and protection for chronic non-communicable diseases that was conducted through telephone interviews directed to the adult population (≥18 years) of the Brazilian FUs [14]. These data were used as proxy variables for individuals aged ≥40 years. Once that it was assumed that the tobacco consumption, the percentage overweight and obesity in both population have similar relationships.
- The *rate of contamination by (HPV)* was determined from the proxy variable rates represented by the results of pathological anatomical examinations of the uterine cervix which were recorded in the Cancer Information System of the Uterine Cervix (procedure 12.012.03–3), being selected the diagnoses of benign lesions with cytoarchitectural alterations compatible with HPV viral action [14].
- The *consumption of pesticides* (tons of active ingredient and similar registered) was obtained from records of pesticides consumption which are available on the website of the Brazilian Institute of Environment and Natural Renewable Resources [15].
- The *percentage of physical inactivity* (adults aged ≥18 years who reported not practicing any physical activity in all domains studied were used as proxy variable for individuals aged ≥40 years), *the socioeconomic status* (illiteracy rate, proportion of population with per capita household income below 1/2 salary, Human Development Index - HDI), *the health care utilization* (proportion of the population that had a medical appointment in the last year, proportion of the population who did dental appointment in the last year, proportion of the population who have never been in any dental appointment, proportion of the population covered by health plan, proportion of population with hospitalization in the last year, performance index of the single health system, percentage of population coverage by family health strategy teams, percentage of population coverage by oral health teams) and the *percentage* of *food consumption in kcal/day* per capita (total food consumption, cereals, meat, dairy products, fish, fruits, vegetables, oils and fats, sugars, soft drinks, alcoholic beverages, ready-to-eat foods and industrial mixtures) were recovered from IBGE website [13].

### Statistical analysis

Univariate exploratory analysis of the spatial data was performed for global spatial autocorrelation investigation of the OC mortality rates in the Brazilian FUs by Moran index I, under the assumptions of normality and randomization [16]. The distribution of values of the Moran index varies between − 1.0 and + 1.0 and it tests areas connected to the greater reality for the studied indicator, than it would be expected in a random pattern. So, spatial autocorrelation measures how much close objects are in comparison with other close objects using Moran’s I index that can be classified as positive, negative and no spatial autocorrelation.

The variables of this study were grouped into six blocks to jointly evaluate the indicators associated with OC mortality by Pearson correlation coefficient (r), where the direction and magnitude of the associations among the independent variables were evaluated through a correlation matrix: 1) habits and lifestyle; 2) sociodemographic indicators; 3) consumption of pesticides; 4) presence of comorbidities; 5) use of health services and 6) food consumption. For this analysis, all variables were standardized with mean zero (0.0) and standard deviation equal to one (1.0), due to their different dimensions, which could impair their inclusion and interpretation in the model.

Spatial multiple regression was performed in the last phase of analysis. The fit quality of the spatial regression model is similar to the traditional linear regression model, verified by residue analysis and also based on the Moran index I [17]. The following criteria were used for the inclusion or withdrawal of the variables of the model: 1) Selection of variable with higher statistical correlation; 2) Inclusion of variables that, when analyzed together, obtained higher F in the simple regression analysis; This inclusion does not prevent that variables from the same block are also included as “adjustment variables”, regardless of their association. 3) Inclusion of variables which once in partial correlation, controlled by modeled variables, showed significant correlation with the dependent variable. The final model exclusion criteria for variables were the *p* value ≥0.05.

Graphical analyzes were performed between standardized residues in the verification of linear regression assumptions, observed and predicted values, and the diagnosis of normality using Q-Q plot plots. The post-tests of Breusch-Pagan and Koenker-Bassett for verification of heteroscedasticity were also applied. The non-spatial autocorrelation of residues was also verified in obtaining final models. The software used was Stata 11.0, SPSS 18.0 and GeoDa 0.95-i.

## Results

**Fig. 1:**
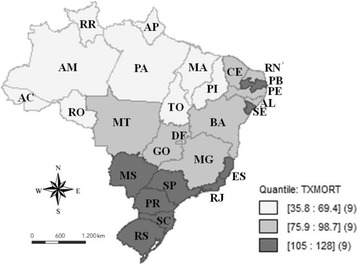
Mortality rates for mouth cancer standardized for the age of 40 years or over per 100,000 inhabitants, in the Brazilian Federative Units, in the period from 2005 to 2014, according to tertile

Figure 1 shows the spatial distribution of OC mortality rates to age ≥ 40 years. It is observed that the highest rates occurred in the South and Southeast region, followed by the Northeast and Midwest regions of Brazil, with the lowest rates being identified in the Northern region.

**Fig. 2:**
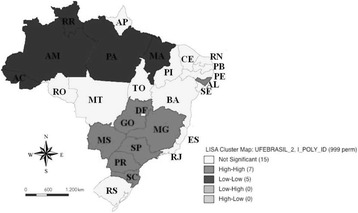
Moran dispersion map for mortality rates from mouth cancer standardized for the age of 40 years or over per 100,000 inhabitants, in the Brazilian Federative Units, in the period from 2005 to 2014

The spatial distribution of this rate was not random, with high spatial autocorrelation (*I* = 0.648; *p*-value = 0.001 for 999 permutations). It was possible to observe the occurrence of spatial autocorrelations of the “high-high” type, indicating the grouping of Brazilian FUs with higher mortality rates due to OC located in the following regions: South (SC and PR), Southeast (SP and MG), Central-West (MS and GO) and Northeast (AL). It was also observed the occurrence of “low-low” spatial autocorrelations, considered as a group with the lowest rate, represented by the States of AC, AM, RR and PA (Northern region) and MA (Northeast region) (Fig. 2).

**Fig. 3:**
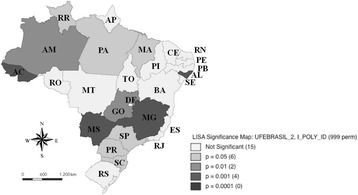
Identification of the occurrence of significant clusters, based on the analysis of the Local Moran index, for mortality rates for mouth cancer for the age of 40 years or over per 100,000 inhabitants, in the Brazilian Federative Units, in the period from 2005 to 2014

Moran Local Statistics I, shown in Fig. 3, is also of great importance for the analysis, since it shows the degree of significance of certain groups. By the Local Spatial Association Indicator (LISA), the states with the highest death rates for OC for age ≥ 40 years were MS, MG and AL. The analysis identified significant sectoral clusters from the LISA. The significance of this indicator in the period under consideration implies that there are positive multidirectional externalities of mortality rates due to OC in some FUs of Brazil.

In the bivariate analysis (Table 1), the consumption of pesticides and similar registered, the proportion of the population that consulted in the last year, the proportion of the population that dentist visited last year, the proportion of the population with a health plan coverage, the average of performance index of the single health system, the percentage of physically inactive adults, the HPV contamination rate, the percentage of consumption of oils and fats, the percentage of soft drink, the percentage of alcohol consumption, the percentage of consumption of ready-to-eat foods and industrial mixtures and the percentage of overweight adults were positively correlated (*p* < 0.05) with mortality rates from OC. There were also negative significant correlations among the proportion of the population who have never been in any dental appointment, the proportion of the population with per capita household income below ½ salary, the HDI mean and the percentage of fish consumption and the mortality rates for OC (*p* < 0.05).

**Table 1:** Correlation matrix among standardized mouth cancer mortality rates for the age group of ≥40 years per 100,000 inhabitants in the Brazilian Federative Units, in the period 2005–2014, and the variables selected in the different blocks of analysis

	ZMC	ZP	ZMA	ZDA	ZNDA	ZHIC	ZISHS	ZPIA	Z½S	ZHDI	ZFC	ZFC	ZOFC	ZRC	ZAC	ZRFIMC	ZOW
ZMC	1																
ZP	.630^**^	1															
ZMA	.468^*^	.273	1														
ZDA	.339^*^	.419^*^	.754^**^	1													
ZNDA	−.522^**^	−.515^**^	−.714^**^	−.844^**^	1												
ZHIC	.763^**^	.612^**^	.658^**^	.629^**^	−.871^**^	1											
ZISHS	.588^**^	.520^**^	.693^**^	.636^**^	−.734^**^	.779^**^	1										
ZPIA	.389^*^	.095	.173	−.135	−.034	.200	.150	1									
Z½S	−.486^*^	−.612^**^	−.525^**^	−.653^**^	.887^**^	−.874^**^	−.747^**^	.029	1								
ZHDI	−.582^**^	.579^**^	.596^**^	.709^**^	−.882^**^	.879^**^	.750^**^	.066	−.935^**^	1							
ZHPV	.859^**^	.519^**^	.377	.178	−.300	.538^**^	.406^*^	.255	−.280	.335	1						
ZFC	−.311^*^	−.378	−.425^*^	−.277	.452^*^	−.443^*^	−.678^**^	.079	.556^**^	−.394^*^	−.284	1					
ZOFC	.210^*^	.509^**^	.182	.055	−.205	.251	.242	−.003	−.344	.233	.261	−.368	1				
ZCR	.644^**^	.580^**^	.344	.544^**^	−.749^**^	.856^**^	.542^**^	.045	−.825^**^	.838^**^	.373	−.228	.144	1			
ZAC	.564^**^	.497^**^	.374	.445^*^	−.696^**^	.763^**^	.623^**^	.148	−.738^**^	.690^**^	.306	−.434^*^	.026	.700^**^	1		
ZRFIMC	.581^**^	.500^**^	.526^**^	.735^**^	−.867^**^	.846^**^	.728^**^	−.030	−.841^**^	.833^**^	.324	−.406^*^	−.045	.837^**^	.807^**^	1	
ZOW	.106^*^	.211	−.066	.221	−.260	.262	.065	.166	−.301	.284	−.123	.121	.009	.460^*^	.390^*^	.299	1

**p < 0,01

*p < 0.05

ZMC: age-standardized oral cancer mortality rate; ZP: consumption of pesticides and similar products (tons of active ingredient); ZMA: proportion of the population who did medical appointment in the last year; ZDA: proportion of the population who did dental appointment in the last year; ZNDA: proportion of the population who have never been in any dental appointment; ZHIC: proportion of the population with health insurance coverage; ZISHS: average performance index of the single health system; ZPIA: percentage of physically inactive adults; Z½S: proportion of population with per capita household income below 1/2 salary; ZHDI: average human development index; ZHPV: HPV contamination rate; ZFC: percentage of fish consumption kcal / day per capita; ZOFC: percentage of oils and fats consumption kcal / day per capita; ZRC: percentage of refrigerant consumption kcal / day per capita; ZAC: percentage of alcohol consumption kcal / day per capita; ZRFIMC: percentage of ready-to-eat foods and industrial consumption mixtures kcal / day per capita and ZOW: percentage of overweight adults (IMC ≥ 25 kg/m^2^)

Table 2 shows the results of the final spatial multiple regression analysis model. The HDI presented a significant inverse association with mortality rates from OC in the study period (p < 0.05). Besides, the proportion of the population who did dental appointment within the last year, the percentage of consumption of oils and fats, the percentage of ready-to-eat foods and industrial mixtures consumption and the percentage of overweight adults were positively associated with mortality rates of t OC (*p* < 0.05).

**Table 2:** Spatial Multiple Regression Model of mortality rates for mouth cancer standardized for age ≥ 40 years, per 100,000 inhabitants and associated factors in the Brazilian Federative Units in the period of 2005–2014

Variables	Coefficients	Standard Deviation	t	*p*-value
Percentage of oils and fats consumption^a^	1.690	0.767	2.198	0.039
Percentage of ready-to-eat food and industrial consumption mixtures^a^	9.233	3.754	2.459	0.023
HDI mean	−1.854	5.741	−3.229	0.004
Percentage of overweight adults^b^	2.598	0.732	3.547	0.002
Proportion of the population who dental appointment within last year	1.339	0.504	2.659	0.015

^a^kcal/day per capita

^b^IMC ≥ 25 kg/m^2^

## Discussion

This study has some limitations inherent to the methodology used, and the possibility of ecological fallacy cannot be excluded since an association observed between aggregates does not necessarily mean that the same association occurs at the individual level [18]. The low construct validity is another possibility that cannot be discarded, since not all outcome explaining variables of the may have been included in the methodology used [19]. In order to reduce some of these limitations, this study has worked with variables available in several national databases and may present differences in quality that are inherent to the use of indirect estimates [20].

The results of the present study show a fairly unequal distribution of mortality rates due to OC (Fig. 1). In order to adjust the differences in the age distribution of the population and consequently a possible confounding effect of the age structure on the mortality rates due to OC in the FUs of Brazil, the direct method of standardization of these rates was used [21]. Since the incidence of this cancer has increased in young adults (< 45 years) in countries with high incidence of this neoplasm in the world [2].

There was a predominance of significant spatial groupings of mortality rates due to OC for age-standardized in the Central-South region of the country (Fig. 2) that it was confirmed by the LISA (Fig. 3). Brazil is a country with continental dimensions, divided into five geographic regions with different demographic, economic, cultural and health conditions and generalized internal inequalities [22]. Considering this context, it is important to highlight that although genetic factors play an important role in carcinogenesis, environmental, behavioral and occupational risk factors play a strong role in this process [23].

The HDI was negatively associated with mortality rates for OC for age-standardized in multiple spatial regression. It has been observed that socially disadvantaged groups tend to have greater contact with several risk factors, as well as poor oral health conditions, nutritional deficiencies and access to health services [24]. Ferlay et al. [25] report that both the crude rate and the age-standardized incidence rate of the OC in the world population are higher in more developed regions, but mortality is higher in less developed areas, which shows social inequality. Significant spatial groupings in the Brazilian FUs that have medium and high human development index were observed (Fig. 2), corroborating with the findings of Borges et al. [26] which reported higher mortality rates in Brazilian capitals with high HDI. It is important to note that both survival and quality of life related to OC depend mainly on its clinical stage at the time of diagnosis and also on access to evidence-based multidisciplinary treatments [27]. This has not been a reality for most of the developing world, since health education and early diagnosis are rare in these countries and most of these tumors are diagnosed late [28].

The proportion of the population who had a dental appointment in the last year was positively associated with death rates from OC in the final spatial model. Early diagnosis is crucial for improving your the survival rates, since this type of cancer has one of the lowest rates of survival over 5 years when compared to the most prevalent carcinomas, among which are included breast cancer, skin, testicles, prostate, uterus and urinary bladder [29]. Therefore, early diagnosis and immediate treatment can significantly reduce the morbidity associated with its therapy and consequently improve overall long-term survival [30], since in most cases its evolution is slow [31]. Its early stage is usually asymptomatic [32] and the most lesions are not diagnosed until they have reached advanced stages despite it was occurring in a part of the body that is readily accessible for early detection [33]. About 50% of oral cancer patients are diagnosed in the advanced stage and make a first appointment with a health professional within 2 months after identifying some sign or symptom, while 20–30% of the patients delay seeking professional help for more than 3 months [33]. The patient’s delay in consulting a health professional can be attributed to the patient’s delay in identifying the signs or symptoms of these cancer or to difficulties in accessing professional care [34]. Additionally, there is a shortage of trained or specialized professionals in oral diagnosis or stomatology acting in the primary and secondary care of the public health system of Brazil, limiting the offer of services of this nature [35]. In addition, access to private and supplementary health services in Brazil is dependent on the individual’s economic status [35].

The percentage of overweight adults remained positively associated with death rates from OC in this ecologic study. Overweight seems to have a mutual relationship with oxidative stress [36]. This in turn is able to fragment DNA, RNA, lipids and protein and consequently interfere in the DNA repair system, contributing to the development of diseases, such as cancer [37].

In these study, the percentage of consumption of oils and fats remained positively associated with mortality rates due to OC [38]. The usual presence of saturated animal fat in the Brazilian diet has been identified as a risk factor for OC and some mechanisms have been proposed to explain the influence of fatty acids on carcinogenesis and include the peroxidation of polyunsaturated fatty acids and subsequent DNA damage; effects on estrogen concentrations and their availability; effects on membrane-bound enzymes that regulate xenobiotic metabolism; changes in cell membranes, resulting in changes in hormone receptors and growth factor; regulation of fatty acids from the production of eicosanoids and subsequent modulation of the immune response; activation of fatty acids from nuclear transcription factors, leading to cell differentiation; modulation of signal transduction pathways by fatty acids, leading to altered gene expression and effects on cell proliferation and apoptosis and inhibition of translation initiation, leading to a decrease in cell proliferation due to the reduction of G1 cyclin synthesis and expression and G1 cell cycle arrest [39].

The percentage of ready-to-eat foods and industrial mixtures consumption has remained positively associated with mortality rates due to OC in the multiple spatial model. The consumption of ready-to-eat foods or fast foods have been associated with increased risk of OC [38]. Furthermore, the consumption of foods, fried, baked or cooked in the microwave should be avoided by increasing the risk of this type of cancer, due to the formation of heterocyclic amines [40]. Other aspects that should be studied in order to prevent this malignant neoplasm are the methods of preparation and conservation of foods because they can collaborate directly or indirectly in the development of certain types of cancer [41].

## Conclusion

This is the first study that analyzed the factors associated to the spatial clusters of mortality due to oral cancer in the Brazilian Federative Units. Although public policies to combat OC have been introduced since the beginning of the twenty-first century in Brazil, this study show a fairly unequal distribution of OC mortality rates that presented inverse association with HDI and direct association with dental appointment in the last year, consumption of oils and fats, ready-to-eat foods and industrial mixtures consumption and overweight these rates. It suggests the need to redirect Brazilian public policies aimed at combating the potential etiological contributors so that these health policies can be permanent and effective.

## Abbreviations

AL: Alagoas State
CAAE: Certificate of Presentation for Ethical Appreciation
DNA: Deoxyribonucleic acid; RNA, Ribonucleic acid
FUs: Federative Units
GO: Goiás State
HDI: Human Development Index
HPV: Human papillomavirus
IBGE: Brazilian Institute of Geography and Statistics
kcal: Kilocalories
LISA: Local Spatial Association Indicator
MG: Minas Gerais State
MS: Mato Grosso do Sul State
OC: Oral cancer
PR: Paraná State
r: earson correlation coefficient
SC: Santa Catarina State
SP: São Paulo State
